# APOBEC mutagenesis and selection for NFE2L2 contribute to the origin of lung squamous-cell carcinoma

**DOI:** 10.1016/j.lungcan.2022.07.004

**Published:** 2022-07-09

**Authors:** Vincent L. Cannataro, Shalley Kudalkar, Krishna Dasari, Stephen G. Gaffney, Heather M. Lazowski, Laura K. Jackson, Isil Yildiz, Rahul K. Das, Bonnie E. Gould Rothberg, Karen S. Anderson, Jeffrey P. Townsend

**Affiliations:** a Department of Biology, Emmanuel College, Boston, MA, USA; b Department of Pharmacology, Yale University, New Haven, CT, USA; c Yale College, New Haven, CT, USA; d Department of Biostatistics, Yale School of Public Health, New Haven, CT, USA; e Yale Cancer Center, Yale University, New Haven, CT, USA; f Department of Pathology, Yale University School of Medicine, New Haven, CT, USA; g Department of Pathology, VACT Healthcare System, West Haven, CT, USA; h Yale Cancer Center Innovations Laboratory, Yale Cancer Center, New Haven, CT, USA; i Department of Molecular Biophysics & Biochemistry, Yale University, New Haven, CT, USA; j Department of Ecology and Evolutionary Biology, Yale University, New Haven, CT, USA; k Program in Computational Biology and Bioinformatics, Yale University, New Haven, CT, USA

**Keywords:** Cancer evolution, Molecular signatures, NFE2L2, APOBEC, Molecular epidemiology

## Abstract

Lung squamous-cell carcinoma originates as a consequence of oncogenic molecular variants arising from diverse mutagenic processes such as tobacco, defective homologous recombination, aging, and cytidine deamination by APOBEC proteins. Only some of the many variants generated by these processes actually contribute to tumorigenesis. Therefore, molecular investigation of mutagenic processes such as cytidine deamination by APOBEC should also determine whether the mutations produced by these processes contribute substantially to the growth and survival of cancer. Here, we determine the processes that gave rise to mutations of 681 lung squamous-cell carcinomas, and quantify the probability that each mutation was the product of each process. We then calculate the contribution of each mutation to increases in cellular proliferation and survival. We performed in vitro experiments to determine cytidine deamination activity of APOBEC3B against oligonucleotides corresponding with genomic sequences that give rise to variants of high cancer effect size. The largest APOBEC-related cancer effects are attributable to mutations in PIK3CA and NFE2L2. We demonstrate that APOBEC effectively deaminates NFE2L2 at the locations that confer high cancer effect. Overall, we demonstrate that APOBEC activity can lead to mutations in NFE2L2 that have large contributions to cancer cell growth and survival, and that NFE2L2 is an attractive potential target for therapeutic intervention.

## 1. Introduction

Apolipoprotein B mRNA editing catalytic polypeptide-like (APOBEC) protein activity has been implicated as a mutagenic agent within several cancers [1–3]. APOBEC proteins catalyze a cytidine to uridine (C → U) conversion in single-stranded DNA, and their catalysis of cytidine deamination of viral DNA is thought to provide a protective mechanism at the cellular level against viral infection [4]. APOBEC3B, a member of the APOBEC protein family, is a significant contributor towards APOBEC mutagenesis [5,6], and is upregulated in many cancer types—especially cancers originating within tissues that are exposed to pathogens, e.g. lung squamous-cell carcinoma (LUSC) and adenocarcinoma, bladder, breast, head and neck, and cervical cancers.[1,2,7,8] Furthermore, APOBEC proteins efficiently deaminate deoxycytidines neighboring DNA damage associated with reactive oxygen species and tobacco, and thus APOBEC mutagenesis is particularly relevant to lung tissue. [9]. APOBEC proteins induce distinct mutational patterns within the genome [10], and thus their mutational signature can be deconvolved from total tumor mutation burden [11]. These signatures have been associated with many cancer types, including LUSC [2,3,8,12–15]. However, the majority of single-nucleotide variants within cancer are “passengers” and do not directly contribute to the cancer phenotype of increased cellular division and survival [16,17]. APOBEC mutagenesis itself has not been directly associated with LUSC cell proliferation. Therefore, the relation of APOBEC activity to the cancer phenotype within LUSC depends on the probability that relative increases in cellular proliferation are attributable to APOBEC mutational processes. This probability can be calculated via analysis of the relative contribution of variants to cell lineage proliferation, the trinucleotide context of these variants, and the proportion of total mutational weight attributable to APOBEC activity.

At a biochemical level, APOBEC3B (and other APOBECs such as APOBEC3A and APOBEC3H) is a cytidine deaminase that acts upon single stranded-DNA as a substrate. The physiologically relevant gene substrates for APOBEC3B that change molecular mechanisms, give rise to cancer phenotypes and may be important targets for pharmaceutical development are still being explored. A number of genetic and biochemical studies have established that cytidine deaminase exhibits a local substrate sequence preference for cytidine sites that are preceded with thymidine, such that TC is the base recognition sequence within the target gene [2]. The consequent TU dinucleotide can give rise to two types of mutations. In one case, the uracil can base pair with adenine, undergoing subsequent thymidine incorporation to result in C → T transitions. This first case represents the proposed etiology of the majority of mutations present within the COSMIC single-base-signature 2. In another case, the uracil can be recognized for excision by uracil nucleoside glycosylase to create an abasic site, whereupon C → G transversions can occur via error-prone guanine incorporation. This latter case is putatively the mechanism underlying the majority of mutations present within COSMIC single-base-signature 13 [7]. The goal of the current work was to evaluate the increases in cellular proliferation that are attributable to tumor-prevalent mutations arising from APOBEC mutational processes, and to confirm that the sites identified would be plausible enzymatic target substrates for cytidine deamination by APOBEC3B.

Initial LUSC tumor sequencing studies identified TP53, CDKN2A, PTEN, PIK3CA, KEAP1, MLL2, HLA-A, NFE2L2, NOTCH1 and RB1 as drivers [18,19]. Subsequent studies have identified EGFR [20], NSD3 [21], and KEAP1 and NFE2L2 [22] as LUSC drivers. Of these driver genes, driver mutations of PIK3CA [23] and KEAP1/NFE2L2 [24] have already been demonstrated to be associated with APOBEC signature enrichment, and PIK3CA variants have furthermore been demonstrated to possess high APOBEC-mutation-driven effect sizes in head and neck cancer [25]. To quantify the strength of selection on LUSC driver mutations in these genes and to guide experimental investigation, we sequenced whole exomes of a large set of LUSC tumors. We analyzed these data—along with publicly available tumor sequence data—to calculate effect sizes[26] for mutations that drive LUSC tumorigenesis. Deconvolving mutational signatures within each tumor, we evaluated which variants are attributable to APOBEC based on the attributable component of COSMIC Signature 2 and 13. We quantified the degree to which effect sizes associated with each driver variant are attributable to known mutational processes. Lastly, we focused on the LUSC driver gene with the largest APOBEC-mutation-driven cancer effect sizes, apart from PIK3CA. To validate a putative role of APOBEC3B in originating those mutations, we assessed the activity of one of the APOBEC proteins (APOBEC3B) with an in vitro biochemical assay on the nucleotide substrate corresponding to its highly mutated driver sites.

## 2. Materials and methods

### 2.1. Sourcing of tumor samples

Fresh tumor samples (*n* = 59 from 59 patients) snap-frozen in RNALater and matched buffy-coat germline samples stored within liquid nitrogen were obtained from the Yale Cancer Center Lung SPORE Biorepository (YCCLSB). Each of these tumors were from LUSC cancer patients known to be smokers. A further 22 tumor samples with matched normal samples from adjacent tissue were obtained from the West Haven Veterans Administration Medical Center, West Haven, CT (VAMC), selecting all non-small cell squamous cell lung cancer tumors with sufficient tissue for sequencing.

### 2.2. Additional data sources

LUSC whole-exome somatic mutation data was obtained from two public sources: mutations from 492 tumors were obtained from the National Cancer Institute Genomic Data Commons (GDC) [27], and mutations from a further 108 tumors were obtained from the Yale-Gilead (YG) collaboration [26]. Pre-processed GDC variants were downloaded in MAF format from portal.data.gov, and converted to Genome Reference Consortium Human Build 37 (GRCh37) using the liftover package [28]. YG variants and the mutation calling procedure are described in Choi *et al.* [29]. YCCLSB and VAMC cohort variant calls (both unfiltered calls, and the filtered calls used for analysis) are provided in a SYNAPSE repository at https://doi.org/10.7303/syn31770233.

### 2.3. Variant calling and filtering

#### VAMC cohort:

Whole-exome sequencing was performed on the 22 pairs of tumor and matched normal tissue samples. Reads were aligned to human genome reference GRCh37 (hs37d5) using bwa mem (v0.7.10), sorted with SAMtools (v1.2), then duplicates were marked with Picard tools (v1.118). GATK (v3.2-2) was used to identify and realign around indels (RealignerTargetCreator, IndelRealigner), and to perform base quality score recalibration (BaseRecalibrator). MuTect2 (v2.7-1) was used to call short variants (with normal ALT allele caps set at 6 reads, 10% of total reads, and with a maximum Q-score sum of 200), and IndelGenotyper (v36.3336) was used to identify indels. Variants were annotated using vcf2maf (v1.6.21), removing those labeled as ’common variant’. Variants were also removed if present at over 0.04% in any gnomAD subpopulation; had tumor VAF < 5%; had normal VAF ≥ 5%; had fewer than 5 ALT reads in tumor; had ≥5 ALT reads in normal; were present in a Yale panel of normals; were marked as an off-target variant (with a Variant Classification of Intron, 3′Flank, 5′Flank, or IGR); or were in genes known to be problematic in hg19 (PDE4DIP, CDC27, MUC4, DUX4, HYDIN, PRIM2) [30]. The variants that remained were used in our downstream analysis.

#### YCCLSB:

Whole-exome sequencing was performed on matched tumor and normal samples using the Ion Torrent Proton platform as described within Rothberg et al. [31]. Variants were called using a stand-alone version of Torrent Variant Caller (TVC), with a high-stringency setting [32] to minimize false-positive calls. Subsequently, a series of additional QC steps were performed outside Ion Torrent Suite for identifying high-confidence somatic variants. First, variants that are not reported in COSMIC (build from 05/2017) that mapped to known human germline variants from 1000-genome project and dbSNP (build from 05/2017) were dropped. Next, variants that are not reported in COSMIC (build from 05/2017) that did not differ significantly in allele frequency between matched normal and tumor (*P* > 0.05 from Fisher’s exact test) were excluded. Next, the tumor reads were reanalyzed using bam-readcount and analogous filtering metrics to those used by the VarScan2 somatic calling pipeline [33] were applied to filter out false positive calls due to mapping and base-calling errors etc. Furthermore, empirical filters were applied to remove spurious somatic calls specific to amplicon sequencing, such as mispriming and amplicon bias. A homopolymer length-based filter was applied to reduce false-positive multi-nucleotide variants and indels. Finally, somatic mutation calls were compared to our in-house “panel of normals”, a reference of all the variants called across all of the germline samples previously sequenced in the lab to serve as a final filter for germline variants as well as base-calling errors.

### 2.4. Calculating mutation weight

The relative weights of each mutational signature contributing to total mutation burden were deconvoluted by the deconstructSigs package [34]. The deconstructSigs package takes an input data frame *T* of trinucleotide contexts for substitutions in each tumor and a set of known signatures *S*, and calculates the reconstructed tumor sample mutation matrix *R* = *T*−(*S* × *W*) by using a forward selection process to optimize weight *W* by minimizing the sum-squared error between *T* and *S*. Prior to calculation of mutation weights, we removed all variants recurrently substituted among tumors to minimize trinucleotide biases introduced by variants not drifting to fixation [26]. The mutational signatures that were discoverable were the previously detected LUSC signatures within the COSMIC v3.2 signature set [11]. Signatures previously deemed to be sequencing artifacts were removed and remaining weights were normalized to be the proportion of total detected weights in that tumor.

### 2.5. Calculating proportionate attributable effect size

Proportionate attributable effect sizes were calculated using a modification of our previous method [25]. Briefly, the effect size of variants were calculated using the R package cancereffectsizeR version 2.6.1. We assumed that substitutions fixed in accord with a Poisson distribution at the rate mutations arise *μ* multiplied by their cancer effect size *γ*. Unlike a previous analysis [26], we calculated the effect size more precisely for every variant by maximizing the likelihood function.

$$L(\gamma |\mu_{1},\ldots ,\mu_{M},\ldots ,\mu_{Z})=\prod_{i=1}^{M}1-e^{-\mu_{i}\gamma }\times \prod_{i=M+1}^{Z}e^{-\mu_{i}\gamma }$$

where*μ_i_*, 1⩽i⩽Z, is the rate of mutation to variant *k* for this tumor, and where *M* and *Z* are defined such that the variant is present in tumors and there is an absence of any same-gene variants in tumors*M* + 1…*Z*. We excluded tumors with other variants in the same gene from the latter group due to the likelihood of reduced selection for subsequent same-gene mutations in these tumors. Each tumor-specific mutation rate was calculated by extracting the mutation rate in each trinucleotide context of each variant from the tumor-specific mutational signature weights, then convolving it with the gene-specific mutation rate as in Cannataro et al. [26]. To obtain the attributed effect size, for every tumor with recurrent variants, the effect sizes were multiplied by the probability that each mutational signature contributed to that variant, given the variant’s trinucleotide context and the mutational weights within the tumor calculated above, and these attributed effect sizes were renormalized such that each is the proportion of total attributed effect size within the tumor to obtain the proportionate attributable effect size. The bioinformatic pipeline used to complete this analysis and generate the figures is available at https://github.com/Townsend-Lab-Yale/LUSC_APOBEC_NFE2L2_ms/.

### 2.6. Assessing APOBEC3B deamination of NFE2L2 substrates:

Recombinant full-length APOBEC3B with a N-terminal maltose binding protein was expressed and purified from *E. coli* as in Sasaki et al. [35]. A representative purification scheme for A3B and protein gel are shown in Supplemental Fig. 1. For deamination assays, a novel assay, DRONE [35] was employed. This assay was previously developed in our lab to allow direct detection of the cytidine deamination product containing the corresponding uridine using Ultra High Performance Liquid Chromatography (UHPLC) without the need for further process using uracil DNA glycosylase and sodium hydroxide. In this reaction, 5 μM of APOBEC3B was incubated with 1 μM of oligos in a reaction buffer consisting of 50 mM Tris pH 8.0, 100 mM NaCl, 0.1% Triton X-100, and 1 mM DTT [35]. Upon quenching with phenol chloroform, the collected aqueous layer was dialyzed for 3 h against water. The dialyzed samples were processed by UHPLC to monitor the cytidine to uracil conversion using a Poroshell 1.9 μm C18 column, a mobile phase consisting of the ion pairing HFIP-TEA buffer system (Buffer A: 400 mM hexafluoroisopropanol (HFIP), 15 mM trimethylamine (TEA); Buffer B: 50% Buffer A, 50% methanol). The optimal separation was achieved at 45% Buffer B and at 60 °C. A series of control oligonucleotides (oligos) corresponding to either substrates or cytidine deaminated products containing uridine were run on UHPLC to determine the individual chromatographic retention times to allow assignments of the products formed in the enzymatic reaction with APOBEC3B.

All DNA oligonucleotides were ordered high-pressure liquid chromatography-purified from Integrative DNA Technologies. The test oligonucleotide sequences were designed based on the *NFE2L2* sequence. Noncoding- and coding-strand oligos were designed to include the R34 site, which has been attributed to high cancer effect [26]. The test oligonucleotide sequences in which the possible cytidine deamination sites containing the TC recognition sequence is underlined as shown below.

Coding strand (25-mer): 5′-TTGGAGTAAGTCGAGAAGTATTTGA-3′.

Noncoding strand (22-mer): 5′-AATACTTCTCGACTTACTCCAA-3′.

## 3. Results

### 3.1. APOBEC mutagenesis is among the major contributors to genomic mutation in LUSC

**Fig. 1.**
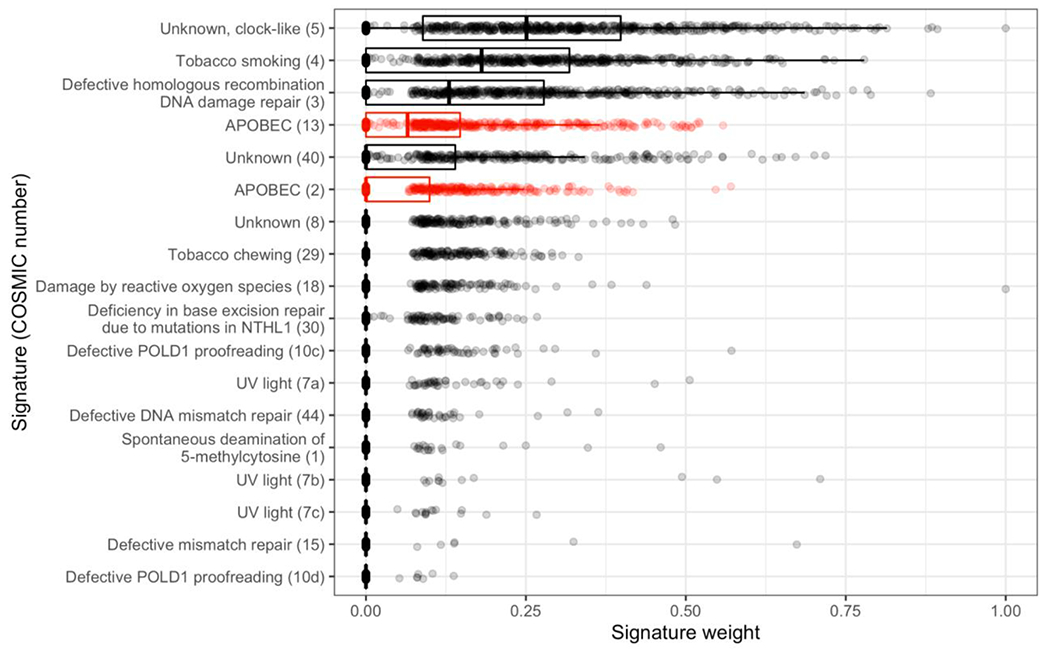
Lung squamous-cell APOBEC (red) and non-APOBEC (black) carcinoma signatures and their weights within our dataset, sorted by descending mean signature weight. Signature weights associated with each tumor (*n* = 681) are plotted and overlaid with a box plot summarizing the distribution across tumors for each signature. (For interpretation of the references to colour in this figure legend, the reader is referred to the web version of this article.)

There are 16 COSMIC mutational signatures that have been previously detected as sources of mutagenesis within LUSC [11]. All of these 16 signatures are detected within our samples. Among these signatures, the two signatures attributable to APOBEC activity have the fourth-highest (Signature 13) and sixth-highest (Signature 2) mean signature weights (Fig. 1). When accounting for the APOBEC signatures as a combined signature, the combined signature has the fourth-highest mean signature weight.

### 3.2. APOBEC mutagenesis is a significant contributor to total cancer effect size in many LUSC tumors

**Fig. 2.**
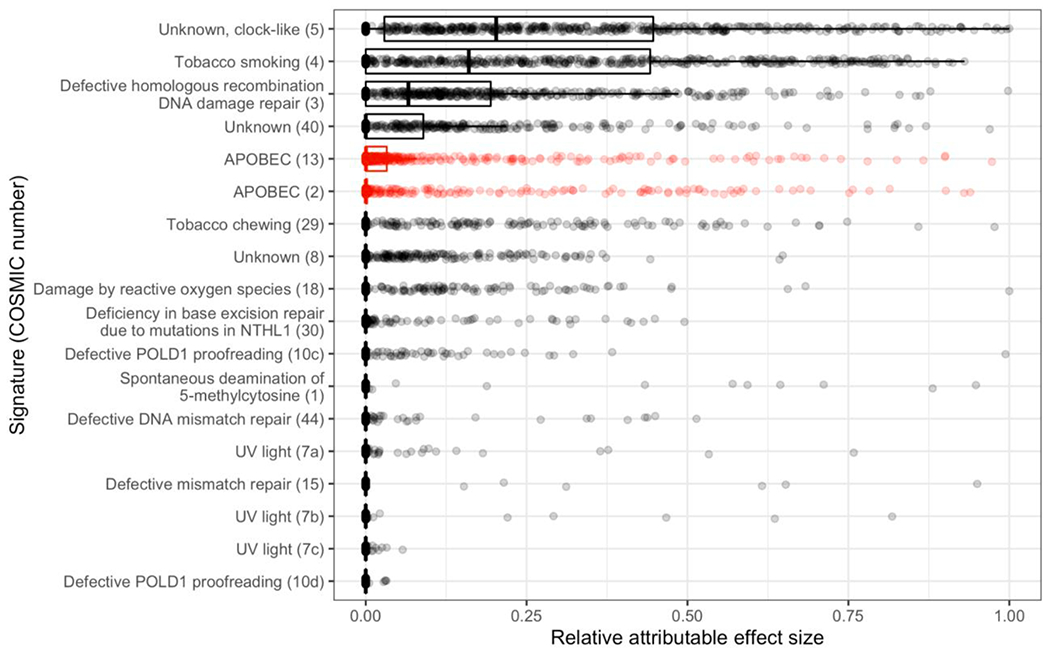
The relative cancer effect size attributable to each APOBEC (red) and non-APOBEC (black) mutational signature in LUSC, in order of descending mean relative attributable effect size. (For interpretation of the references to colour in this figure legend, the reader is referred to the web version of this article.)

To evaluate the importance of APOBEC contribution not only to total mutation burden but also to the mutations that confer cancer phenotype to cells, we quantified and summed per tumor the effect sizes of recurrent variants—a measure of the effect of variants on cancer growth and survival based on how prevalent the variant is among tumors relative to its expected prevalence if mutations did not affect cell division rate and survival. Relative attributable effect sizes can be calculated using per tumor effect sizes in consideration with per tumor signature weights, revealing the relative contribution of mutagenic sources to cancer growth. APOBEC signatures are attributable to the fifth- and sixth-highest mean percentage of the total effect size conferred by recurrent variants within tumors, only surpassed by the common signatures mentioned previously (Fig. 2).

### 3.3. Variants within PIK3CA and NFE2L2 have high cancer effect and are attributable to APOBEC mutational processes

**Fig. 3.**
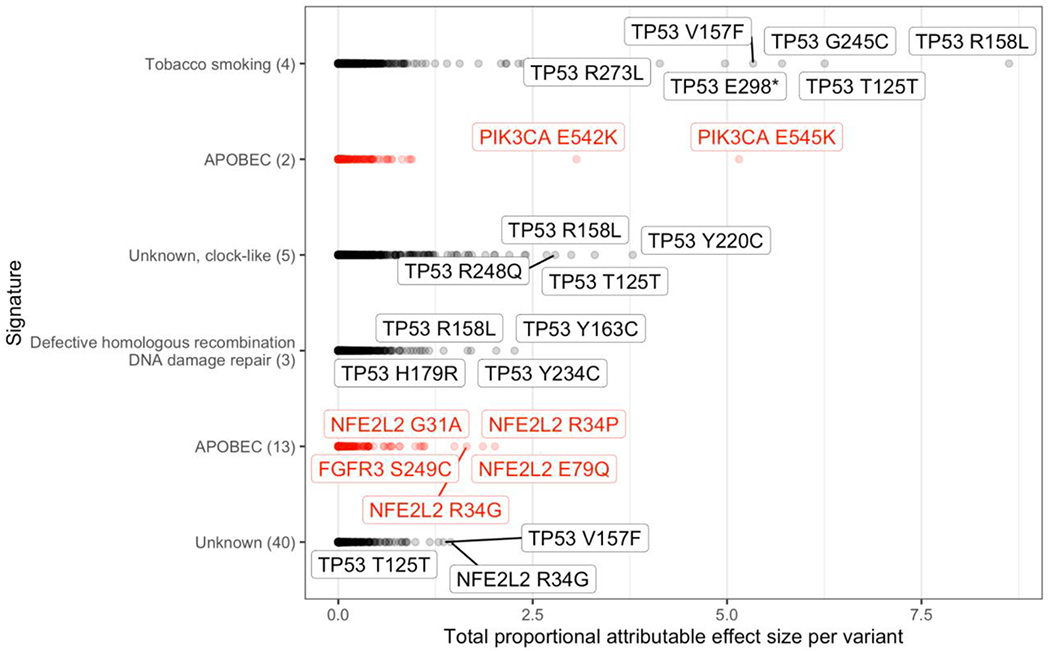
Proportional attributable effect sizes for APOBEC (red) and non-APOBEC signatures. Several variants within NFE2L2 contribute high cancer effect within individual tumors that is attributable to APOBEC Signature 13, and APOBEC Signature 2 contributes high cancer effect via variants in PIK3CA positions 542 and 545. (For interpretation of the references to colour in this figure legend, the reader is referred to the web version of this article.)

Numerous variants within TP53 that are attributable to signatures associated with tobacco smoking, clock-like processes, defective homologous recombination DNA damage repair, and unknown signature 40 are among the variants conferring the highest proportional effect size within the most people (Fig. 3). Two variants within PIK3CA—E545K and E542K—have the highest proportional effect size that is attributable to APOBEC signature 2. Previously, APOBEC3B was demonstrated to deaminate these two sites in *PIK3CA* in vitro [25]. Several variants within NFE2L2, including R34P, E79Q, G31A, and R34G, are the variants with the highest proportional effect size attributable to APOBEC Signature 13. Indeed, activating mutations in *NFE2L2* have recently been discovered to commonly co-occur with *PIK3CA* amplification and mutation in multiple squamous-cell carcinomas including lung, and cooccurrence contributed to greater sensitivity to knockout of either gene in cell lines. [36].

### 3.4. APOBEC3B efficiently deaminates NFE2L2 DNA in vitro

**Fig. 4.**
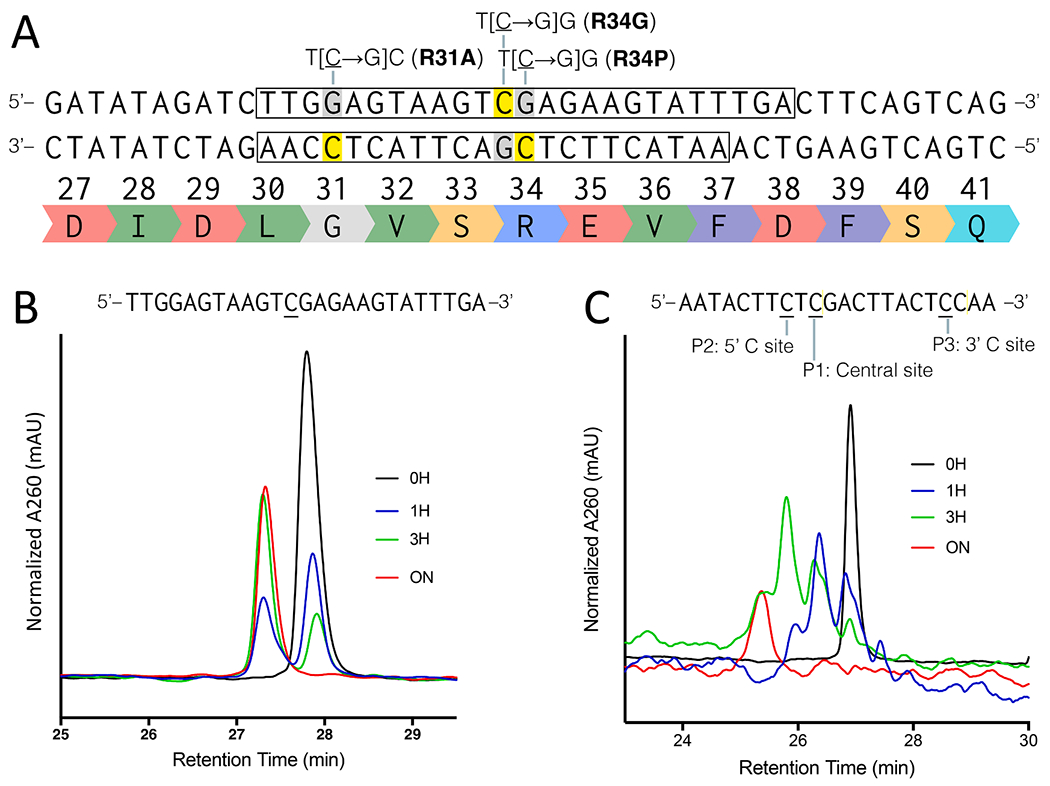
Deamination kinetics of NFE2L2 substrates. (**A**) Schematic for the two complementary sequences for NFE2L2 around mutational hotspot encoding amino acid R34 and possible activating variants. The boxes denote the coding 25-mer and noncoding 22-mer oligo substrates used (**B**) Deamination of the coding strand of the 25-mer NFE2L2 substrate with one targeted cytosine by APOBEC3B. Note that sequence is displayed in a 5’ to 3’ orientation.(**C**) Deamination of noncoding strand of 22-mer NFE2L2 substrate with three targeted cytosines. In each case, the amount of deaminated uridine product formation for the coding and noncoding strands were examined over a period of 1 h to 24 h. H, hour; ON, overnight (24 h).

While the overall ssDNA shape and topology encountered by APOBEC3B in vivo is complex, biochemical studies with shorter oligonucleotide substrates based upon local sequence preferences can provide insights into the molecular mechanism of enzyme catalysis. In previous biochemical studies, we had examined a single-stranded oligomeric substrate incorporating the PIK3CA local TC sequence whose mutation would give rise to the E545K and E542K variants characteristic of helical domain mutations attributed to an APOBEC signature. This previous study demonstrated the ability of APOBEC3B to deaminate a 25-mer oligomeric substrate containing these two TC sites representative of the PIK3CA gene sequence at rates in the range of 0.007–0.023 min^−1^ [33]. The two TC sites corresponding to the E542K and E545K mutants were deaminated in a sequential manner. The site at which the E542K mutation occurs was deaminated first, followed by deamination of the site at which the E545K mutation occurs. In the current study, a similar experiment was carried out to confirm that oligomers containing the relevant NFE2L2 sequences at the amino-acid 34 position could be deaminated. In this experiment the single-stranded oligonucleotide substrates (coding 25-mer and noncoding 22-mer strands, Fig. 4A) were designed based upon the sequence corresponding to translated amino acid 31–38, encompassing the R34 variants of NFE2L2. As described in the methods section, the cytidine deamination activity of APOBEC3B against these substrates was performed using previously published protocol [35]. The rate of deamination was determined by incubating APOBEC3B with substrate oligonucleotides containing the preferred sequence of deamination (NTCN, in which the deaminated cytosine is underlined) for the coding 25-mer and noncoding 22-mer strands from 1 h to overnight (Fig. 4B and Fig. 4C, respectively). After assignment of each peak based on UHPLC runs of substrate and product oligonucleotide individually and mixed together, we found that all the deamination sites were efficiently deaminated by APOBEC3B in a time dependent manner (Fig. 4B–C). In the case of the noncoding 22-mer, as shown in Fig. 4C, we were able to detect sequential deamination events (denoted P1 for central C, P2 for the 5′ C, and P3 for the 3′ C) shown as separating eluting peaks off of the UHPLC column (Fig. 4C), in a manner similar to our earlier studies with the PIK3CA substrate that contained 2 possible TC sites. In the current study, the resulting peaks showed a time-dependent accumulation of a singly deaminated intermediate and its subsequent depletion to form the double and triple deaminated product. In each case, with the coding 25-mer and noncoding 22-mer, the rates of cytidine deamination (0.007 and 0.011 min^−1^) were similar to those for the PIK3CA substrates.

## 4. Discussion

Here we have demonstrated that APOBEC mutagenesis is among the major contributors to genomic mutations in LUSC. Moreover, the mutations induced by APOBEC confer survival or proliferation benefit to LUSC cell lineages. The mutations commonly attributable to APOBEC mutagenesis that make the greatest contribution to cancer proliferation and survival in the most LUSC tumors are PIK3CA mutations such as E545K and E542K, a result that has previously been demonstrated only for head and neck tumors [25]. Moreover, APOBEC-driven mutations to NFE2L2 are also substantial contributors to cancer proliferation and survival, including R34P, E79Q, G31A, and R34G. Biochemical analysis demonstrated that the APOBEC3B protein efficiently deaminates NFE2L2 in vitro at amino-acid position 31 and 34; other APOBEC proteins may have this functionality as well. Our biochemical analysis also demonstrated that the TC at amino acid position 35 (**P2,** Fig. 4C) is deaminated by APOBEC3B. This mutation has not been observed in tumor sequencing data, suggesting that it does not confer an advantage to cellular division or survival, and is not selected to fix within evolving tumor cell populations.

**Fig. 5.**
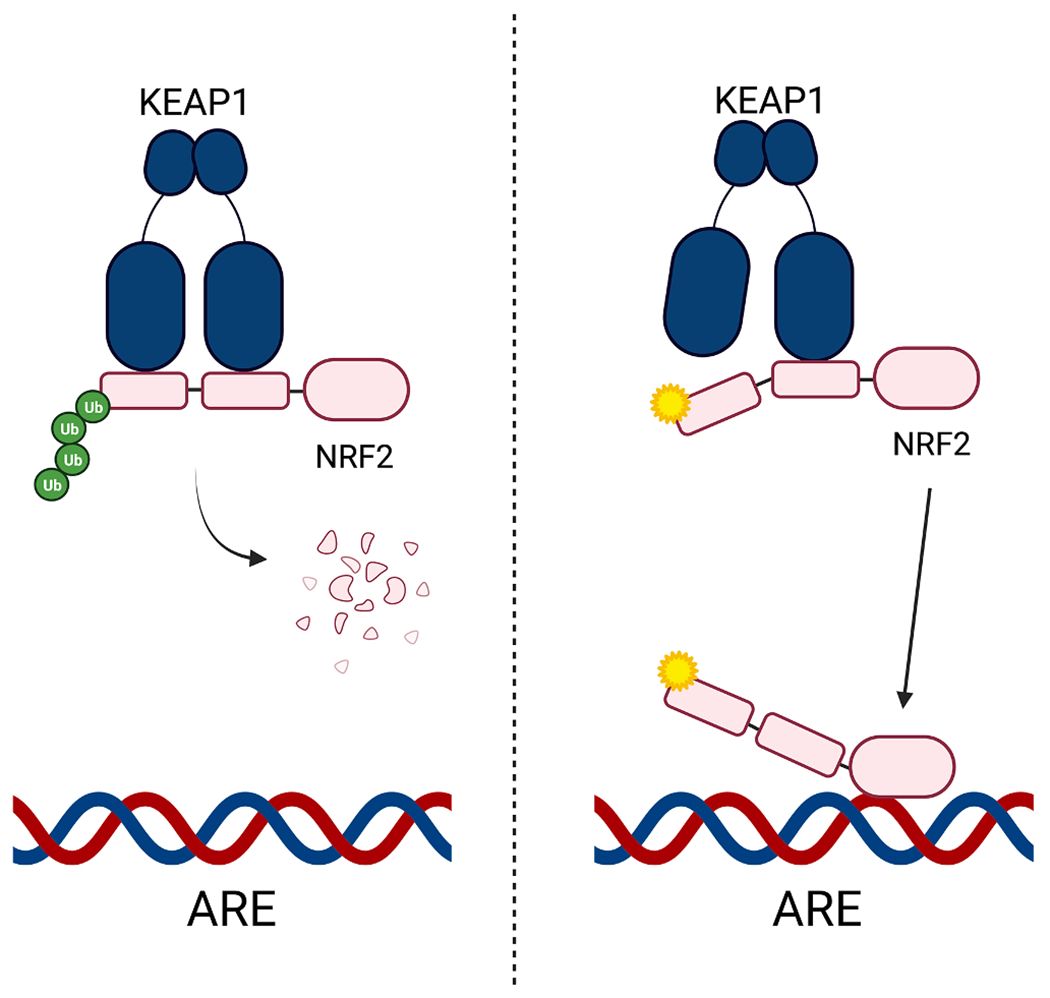
The Nrf2-KEAP pathway to regulate oxidative stress and the nuclear antioxidant response element (ARE) under normal cellular conditions (left) and with Nrf2 activating mutations that lead to dysregulation (right). Figure modeled after Goldstein et al. [39]. Created with BioRender.com.

Historically NFE2L2 has received little attention as a target for therapeutic development [37], perhaps because of its relatively low mutation rate and therefore low prevalence in sequenced tumors. The NFE2L2 gene encodes for the Nrf2 transcription factor, which has been previously demonstrated to be a significantly mutated gene in cervical cancer [38]. Indeed, many of the variants found in cervical cancer sequences conform to the APOBEC mutational signatures [38]. NFE2L2 is a part of the Nrf2-KEAP pathway and one of the mechanisms responsible for regulating cellular response to oxidative stress (Fig. 5). In this pathway, the KEAP protein binds Nrf2 and ubiquitinates it for targeted degradation to control the nuclear antioxidant response element (ARE) in the nucleus [39]. Mutations in Nrf2 decrease binding to KEAP and lead to dysregulation found in a number of cancers including lung cancer. However, recent studies have shown that several mutations in NFE2L2 give rise to activating mutant forms of the Nrf2 that no longer interact with KEAP and lead to proteins with a much longer cellular half-life [40,41]. In particular, arginine 34 is the most frequently mutated and has been suggested to be involved in tumorigenesis and/or cancer progression [40]. These previous studies have shown that the R34P, R34G, and R34Q mutations lead to expression of mutant forms of Nrf2 that are no longer substrates for KEAP and are not targeted for ubiquitination and degradation [40].

NFE2L2 variants have been previously observed at a frequency of around 19% in LUSC samples [18,22], and could be an attractive option for the development of targeted [42] or immunotherapy. NFE2L2 is found mutated in tandem with other specific driver combinations not only in LUSC, but also in head-and-neck, bladder, and esophageal cancers. Therefore, Nrf2 pathway inhibition may interact epistatically with other driving variants to inhibit cancer growth [43]. Therapeutic options providing strong inhibition are highly desirable as NFE2L2-mutant lung cancers have historically had a poor prognosis. The poor prognosis has been traced at least partly to a lack of response of NFE2L2-mutant NSCLC to radiotherapy [44] and to second- and third-line chemotherapy [45]. This lack of responsiveness, in turn, has been attributed to resistance mediated by the mutant-Nrf2-pathway [46–48]. In contrast, immunotherapy has been shown to benefit prognosis and survival in NFE2L2-mutant NSCLC [22] Targeted therapy against NFE2L2 could be applied combinatorially with immunotherapy, and may show strong benefit, based on high cancer effect of the mutations and the potential of abrogation of mutant gain-of-function to reintroduce sensitivity to chemotherapy [46]. Few targeted therapies against NFE2L2 have as yet been developed. Nevertheless, one phase-II trial is ongoing for a targeted therapy against stage 4 or recurrent NFE2L2-mutant LUSC [49]. Other approaches undergoing preclinical testing have been centered around modulation or inhibition of NFE2L2 expression [46,50]. One potentially promising therapeutic strategy would be to selectively degrade mutant forms of Nrf2, which is known to exhibit a much longer cellular half-life [40]. For example, a proteolytic targeting chimeric molecule (PROTAC) strategy has been used to selectively degrade BRAF variants associated with tumorigenesis [51].

In this study, we focused on APOBEC mutagenesis, a commonly observed process in lung squamous cell carcinoma, to quantify the contributions of mutations from this process to cancer growth. However, the methodology is broadly applicable across cancer types and mutational processes. Discernment of mutagenic signatures from sequencing data and quantification of effect sizes for mutations provides the opportunity to identify high-effect, possibly targetable mutations associated with specific mutagenic processes and to verify those associations biochemically. Future applications of this approach can contribute to our understanding of many distinct etiologies of cancers and illuminate the search for therapeutic targets that promise maximal efficacy.

## Supplementary Material

Supplementary Data 1
