# Supplementary figures and images for: APOBEC mutagenesis and selection for NFE2L2 contribute to the origin of lung squamous-cell carcinoma
Source: Lung Cancer. Author manuscript; Available in PMC 2023 Apr 25. (PMC10126952; doi:10.1016/j.lungcan.2022.07.004)

# MBP A3B Purification

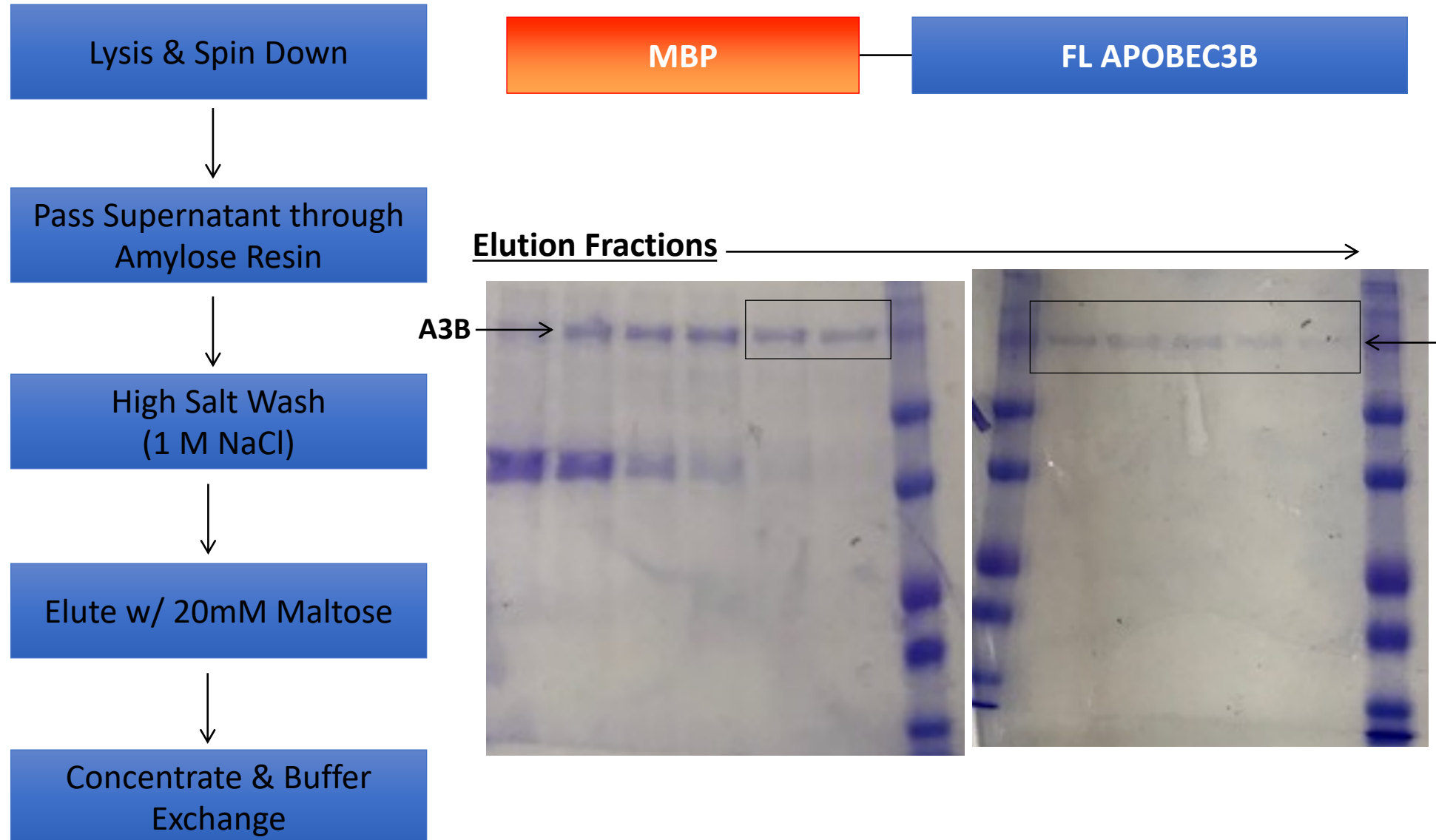

Supplement: Supplementary Data 1 [file NIHMS1887814-supplement-Supplementary_Data_1.pdf]
